# Mutation Analysis of Three Exons of Myosin-Binding Protein C3 in Patients with Hypertrophic Cardiomyopathy

**Published:** 2016-07-06

**Authors:** Maryam Beigom Mobasheri, Mohammad Hossein Modarressi, Cirus Darabian, Ali Akbar Zeinalou

**Affiliations:** 1 *Medical Genetics Department, Faculty of Medicine, Tehran University of Medical Sciences, Tehran, Iran.*; 2 *Cancer Research Center, Cancer Institute, Tehran University of Medical Sciences, Tehran, Iran.*; 3 *Tehran Heart Center, Tehran University of Medical Sciences, Tehran, Iran.*; 4 *Department of Pediatrics, Tehran University of Medical Sciences, Tehran, Iran. *

**Keywords:** *Cardiomyopathy, hypertrophic*, *Death, sudden, cardiac*, *Myosin-binding protein C*, *Mutation*

## Abstract

**Background: **Hypertrophic cardiomyopathy is a genetic disorder with a prevalence rate of 0.2% in the general population. It comes from mutations in sarcomeric proteins. Cardiac myosin-binding protein C3 is one of the critical genes in hypertrophic cardiomyopathy (HCM) and sudden cardiac death, accounting for about 20% of HCM-causing mutations. Genetic testing is recommended in patients with HCM. The aim of the current study was to find possible disease-causing mutations in 3 exons of the gene myosin-binding protein C (MYBPC3) in patients with HCM.

**Methods:** Fifty subjects with documented known HCM were enrolled in the study. The patients were referred to the hospitals affiliated to Tehran University of Medical Sciences between 2008 and 2011. Peripheral blood samples were collected, as well as clinical and demographic data. The nucleotide sequences of the exons number 7, 16, and 18 of MYBPC3, whose relevance to the disease was previously reported, were amplified by polymerase chain reaction. Direct DNA sequencing was applied, and the Chromas software was used to analyze the sequences to find possible disease-causing mutations.

**Results: **The study population comprised 73% male and 27% female patients. The mean age of the patients was 33.9 ± 20.08 years. Family history of sudden cardiac death was reported in 48.2% of the patients. About 79% of the studied subjects had a history of at least 1 other affected relative in their families. Laboratory findings did not show mutations or any nucleotide changes in the sequences of the 3 target exons in the genomic DNA of the studied patients with HCM.

**Conclusion:** The nucleotide sequences of the exons number 7, 16, and 18 of MYBPC3 were not mutated in the 50 studied subjects with HCM.

## Introduction

Familial or sporadic cardiomyopathies are recognized as one of the leading primary cardiac disorders. Hypertrophic cardiomyopathy (HCM) is the most common familial form of cardiomyopathies and affects 0.2% of the general population. As a dramatic event, HCM is the leading cause of sudden cardiac death (SCD) in young people and athletes with no warning signs.^1^ Except for 25%, patients with HCM express none of the disease symptoms.^2^ It is recognized by left ventricular hypertrophy on echocardiography as well as a family history of HCM. The symptom is expressed as chest pain, which may lead to death. Histopathological changes include myocardial hypertrophy, tissue fibrosis, and myocardial disarray. These changes may lead to distorted impulse propagation, nonhomogeneous refractoriness, fibrillation, and SCD. The history of HCM in patients varies from an asymptomatic benign course to a poor prognosis because of heart failure, lethal ventricular arrhythmias, or SCD. As a genetic disorder, HCM is mainly inherited in an autosomal dominant pattern with variable expressions and age-related penetrances.^2^^, ^^3^ Numerous mutations in different sarcomeric genes have been reported. Mutations in myosin heavy chain (MYH7) in 30%, myosin-binding protein C3 (MYBPC3) in 20%, and cardiac troponin T (TNNT2) in 20% of patients constitute the major issues. Mutations in tropomyosin (TPM1) in 5%, cardiac troponin I (TNNI3) in 5%, essential myosin light chain (MYL3) in 5%, regulatory myosin light chain (MYL2) in 5%, and cardiac alpha-actin (ACTC) in 5% of patients with HCM account for the disease-causing mutations as well.^3^

MYBPC is a myosin-associated protein found in the cross-bridge-bearing zone of A bands in the striated muscle.^4^ This crucial component of the sarcomeres and important regulator of muscle function is located at chromosome 11p11.2, having 35 exons which form the coding region of the gene. MYBPC3 is the cardiac isoform of MYBPC and expresses in the heart muscle. In the cardiac isoform, regulatory phosphorylation by cAMP-dependent protein kinase, upon adrenergic stimulation, may be linked to the modulation of cardiac contraction.^4^ MYBPC3 mutations may cause haploinsufficiency and primary increase in calcium sensitivity, which might explain the major features of patients with HCM such as the hypercontractile phenotype and the secondary effects such as myofibrillar disarray, fibrosis, myocardial hypertrophy, and remodeling including arrhythmogenesis.^4^ MYBPC3 mutations are a major causative factor for inherited HCM.^5^^, ^^6^ Patients carrying mutations in this gene have a heterogeneous clinical course (http://www.ncbi.nlm.nih.gov/gene/4607), with some progressing to end-stage heart failure.^6^^-^^8^ The exact cause of this variability is unknown, however.^5^ Some mutations have been reported as severe mutations in different sarcomeric genes which cause SCD or severe disease in young adults.^6^^, ^^9^^, ^^10^ Since mutations in sarcomeric proteins are the primary cause of HCM, molecular diagnosis of the related mutations is important in risk stratification. The morphological and pathological heterogeneity of the disease and the appearance and progression of the symptoms are not straightforward, so the mutation detection of subjects carrying mutations on HCM-associated genes before developing the clinical symptoms is a major issue. For better disease management, it is crucial to be aware of the mutations and devise an appropriate strategy for disease control.^7^^, ^^11^ We studied the DNA sequences of 3 exons of MYBPC3, namely exons 7, 16, and 18, which are known as critical disease-causing exons in some reports^12^^-^^14^ to find possible mutations in Iranian patients with HCM. 

## Methods

This experiment was performed in a case-series design. The study recruited 50 patients with HCM briefly diagnosed on the basic diagnostic criteria, from hospitals affiliated with Tehran University of Medical Sciences. Written informed consent was obtained from all the patients in accordance with the ethical guidelines of the 1975 Declaration of Helsinki, and the study was approved by the local ethics committees.

Peripheral blood was collected from the patients in EDTA-coated tubes. Genomic DNA was extracted using the DNG-plus kit (CinnaGen Inc., DN 8117C, Iran). DNA was amplified by polymerase chain reaction (PCR) to generate the whole sequences of exons 7, 16, and 18 using the appropriate specific primers. The primers were designed to amplify a 528-base-pair (bp) amplicon around the exon 7, as well as 451-bp amplicon around exon 16 and 400-bp amplicon around exon 18 of the gene MYBPC3. The PCR reaction was performed in a total volume of 25 μL containing 30 ng of genomic DNA, 20 pmol of forward and reverse primers, 200 μM of mixed dNTPs, 1.5 mM of MgCl_2_, and 1 U of Taq DNA polymerase (CinnaGen Inc., TA7506C, Iran), with appropriate 10x buffer. The cycling conditions using the ABI thermal cycler 2720 (Applied Biosystems, USA) were as follows: 95 ^°^C for 5 minutes, 33 cycles at 95 ^°^C for 30 seconds, 63 ^°^C for 30 seconds, and 72 ^°^C for 30 seconds followed by a final extension at 72 ^°^C for 5 minutes. Agarose gel, containing GelRed^™^ with a concentration of 1.8%, was used to visualize the PCR products. To purify the PCR products, we used the QIAquick kit-28104 (Qiagen, Hilden, Germany). Direct sequencing reaction was performed in a final volume of 20 μL with 40 ng of the PCR product and 3.2 pmol of the mentioned forward primer using the ABI Prism^®^ 3130 Genetic Analyzer (Applied Biosystems). The sequences were analyzed using the Chromas software program and confirmed with the related normal sequences in the NCBI database.

## Results

Fifty subjects with documented known HCM were enrolled in the study, comprising 73% male and 27% female patients. The youngest patient was 6 months old, and the oldest was 67 years of age (mean age = 33.9 y). Family history of SCD was reported in 48.2% of the patients, and 79.4% of the patients had a history of at least 1 other affected relative in their families. The patients with no family history of HCM or SCD who were > 50 years old were excluded from the study. 

**Figure 1:**
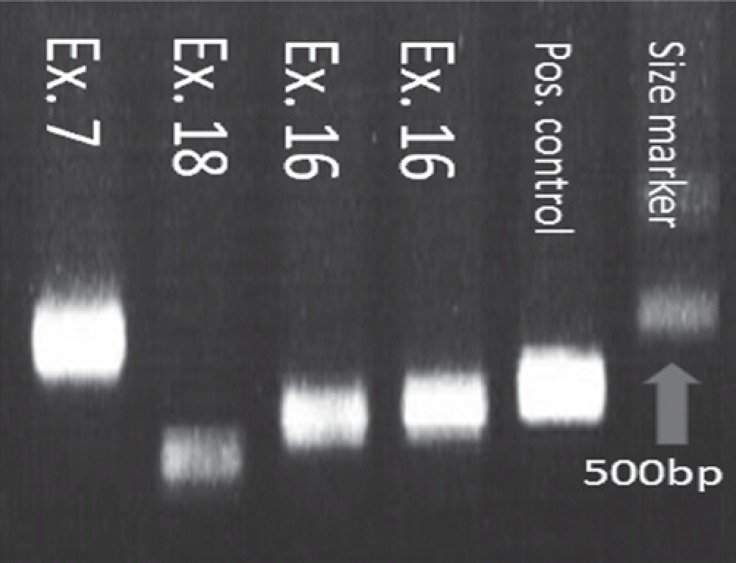
Agarose gel electrophoresis; Lane 1: 528-bp exon 7, lane 2: 400-bp exon 18, lanes 3 and 4: 451-bp exon 16, lane 5: positive control for PCR, and lane 6: 100-bp DNA marker.

The PCR amplification products are shown in Figure 1. The graphs of the amplified sequences were analyzed with the Chromas software and matched with the standard nucleotide sequences in the NCBI database. No deletions, insertions, or other changes were seen in the nucleotide sequences of the genomic DNA of the 3 investigated exons in the studied patients. 

Bp, Base pairs; Ex, Exon; Pos, Positive

## Discussion

Our results demonstrated no mutations in the sequences of the genomic DNA in exons number 7, 16, and 18 of the gene MYBPC3 in the 50 patients with HCM enrolled in the study.

MYBPC3 is a major disease-causing gene in different cardiomyopathies. There are reports of MYBPC3 mutations in hypertrophic and dilated cardiomyophaties.^4^ For instance, the p.Glu441Lys variant identified in exon 16 of the MYBPC3 gene has been reported as a possible pathogenic mutation in Brazilian patients.^13^ However, the underlying molecular mechanisms have yet to be fully elucidated. There have been in-depth studies on MYBPC3 mutations, and genetically altered mouse models have been generated.^4^ MYBPC3 mutations may cause haploinsufficiency and primary increase in calcium sensitivity which is potentially able to explain major features observed in patients with HCM. 

HCM is a heritable disease with autosomal dominant inheritance in affected families. Devising appropriate strategies aimed at identifying those at risk of HCM requires that awareness vis-à-vis disease-causing mutations be raised and HCM be followed among family members through screening the mutation carriers.^15^^, ^^16^

Genetic testing is recommended in patients with HCM in current clinical practice. Nonetheless, various mutation frequencies and clinical manifestations are highly heterogeneous in HCM, both of which limit the use of genetic information in clinical practice. The results of the mutation screening of a study on Spanish patients showed that among 5 HCM-causing genes, MYBPC3 with a frequency of 16% was the most frequently mutated gene followed by the gene MYH7 (8%).^17^ No phenotypic differences were observed between the carriers of the various mutations, which makes it difficult to use genetic information to stratify risk in these patients.^17^

Mutations in MYBPC3 account for about 20% of the total HCM-causing genes.^3^ Nevertheless, there are more than 10 known sarcomeric genes involved in HCM as well as the other regions of MYBPC3, requiring further research with the higher throughput techniques.

## Conclusion

No mutations were observed in the sequences of the genomic DNA in exons number 7, 16, and 18 of the gene MYBPC3, which is a major disease-causing gene in different cardiomyopathies, in the 50 patients with HCM enrolled in the study.

## Notes:

***This paper should be cited as:*** Mobasheri MB, Modarressi MH, Darabian C, Zeinalou AA. Mutation Analysis of Three Exons of Myosin-Binding Protein C3 in Patients with Hypertrophic Cardiomyopathy. J Teh Univ Heart Ctr 2016;11(3):111-114.
